# Supplementary material for: Bedload Transport Monitoring in Alpine Rivers: Variability in Swiss Plate Geophone Response
Source: Sensors (Basel). 2020 Jul 22;20(15):4089. doi: 10.3390/s20154089 (PMC7436244; doi:10.3390/s20154089)
Supplement: Supplementary file 1 [file sensors-20-04089-s001.zip › supplementary material.pdf]

sensors

supplementary material

Bedload Transport Monitoring in Alpine Rivers: Variability in Swiss Plate Geophone Response

Gilles Antoniazza [1,2,*], Tobias Nicollier [1], Carlos R. Wyss [1], Stefan Boss [1] and Dieter Rickenmann [1]

[1] Swiss Federal Institute for Forest, Snow and Landscape Research (WSL), Mountain hydrology and mass movements, Zürcherstrasse 111, CH-8903 Birmensdorf, Switzerland; tobias.nicollier@wsl.ch (T.N.); carlos.wyss@wsl.ch (C.R.W.); stefan.boss@wsl.ch (S.B.); dieter.rickenmann@wsl.ch (D.R.)

[2] University of Lausanne (Switzerland), Institute of Earth Surface Dynamics (IDYST), Géopolis, CH-1015 Lausanne, Switzerland

* Correspondence: gilles.antoniazza@unil.ch; Tel.: +41–21–692–4405 (CH)

Received: 9 June 2020; Accepted: 12 July 2020; Published: date

Content of this file:

Table S1: regression coefficients between the kinetic energy E_k [J] of the impacts and the associated maximum amplitude A_{max} [V] recorded for each SPG at the laboratory experiment and the four investigated measuring stations.

Text S and Figure S: Maximum amplitude A_{max} [V] versus impulse count (> 0.1 V) N_{imp} [-].

Table S2: regression coefficients between the impact maximum amplitude Amax [V] and the associated energy propagation at one and two plates of distance recorded for each SPG at the Avançon de Nant, the Albula and the Naviscence measuring sites.

Table S1. Regression coefficients between the kinetic energy of the impacts E_k [J] and the associated maximum amplitude A_{max} [V] recorded for each SPG at the laboratory experiment and at the four investigated measuring stations. The line 'All' gives the regression coefficient for all the impacts on every sensors taken together at a given measuring station.

	Intercept	Slope	R2
**Avançon de Nant**			
Geo1	10.312	1.198	0.986
Geo2	7.895	1.082	0.992
Geo3	9.910	1.121	0.994
Geo4	7.565	1.112	0.992
Geo5	7.820	1.074	0.992
Geo6	7.411	1.067	0.984
Geo7	7.052	1.149	0.985
Geo8	8.979	1.101	0.986
Geo9	6.823	1.088	0.990
Geo10	8.094	1.091	0.987
All	8.183	1.107	0.991
**Albula**			
Geo2	5.508	1.178	0.986
Geo3	6.844	1.145	0.987
Geo4	6.041	1.133	0.988
Geo5	3.878	1.166	0.972
Geo6	2.290	0.945	0.982
Geo7	1.271	0.881	0.989
Geo8	1.814	0.987	0.963
Geo9	2.439	1.052	0.965
Geo10	6.542	1.142	0.958
Geo11	5.047	1.159	0.989
Geo12	5.659	1.039	0.925
Geo13	4.913	1.009	0.953
Geo14	5.566	1.077	0.966
Geo15	4.944	1.070	0.948
All	4.528	1.077	0.988
**Naviscence**			
Geo1	5.643	1.050	0.976
Geo2	6.247	1.095	0.983
Geo3	4.676	1.064	0.992
Geo4	0.943	0.894	0.950
Geo5	1.821	1.027	0.941
Geo6	2.003	0.963	0.957
Geo7	5.900	1.115	0.985
Geo8	1.019	0.893	0.953
Geo9	2.690	0.989	0.969
Geo10	4.417	1.019	0.964
Geo11	4.067	0.954	0.973
Geo12	6.582	1.088	0.958
All	3.867	1.030	0.99
**Riedbach**			
Geo1	3.991	1.129	0.977
Geo2	3.733	1.120	0.970
Geo3	3.705	1.119	0.979

Geo4	4.394	1.190	0.972
Geo5	4.700	1.171	0.968
Geo6	3.615	1.116	0.985
Geo7	3.491	1.192	0.975
All	3.963	1.144	0.984
**Laboratory**	11.018	1.121	0.977

Text S: Maximum amplitude A_{max} [V] versus impulse count (> 0.1 V) N_{imp} [-].

The maximum amplitude A_{max} and the impulse count N_{imp} are linked by a power law function with exponents close to 0.3 for the impact experiment developed in this paper, at all three measuring sites (Figure S1). The relations are not directly comparable to previously reported similar relations in the literature because of difference in the conditions of the experiment [1]. The above relationships are important to understand the link between A_{max} and N_{imp}, notably the way the energy propagates across plates (Figure 10 in the main document). Results in Figure S2 show that the Avançon de Nant measuring station records about 30% more impulses for a given magnitude (energy) impact than the other two stations, potentially due to the lower rate of attenuation of the propagated energy across plates at this site, which leads to a greater number of impulses counted on non-impacted plates.

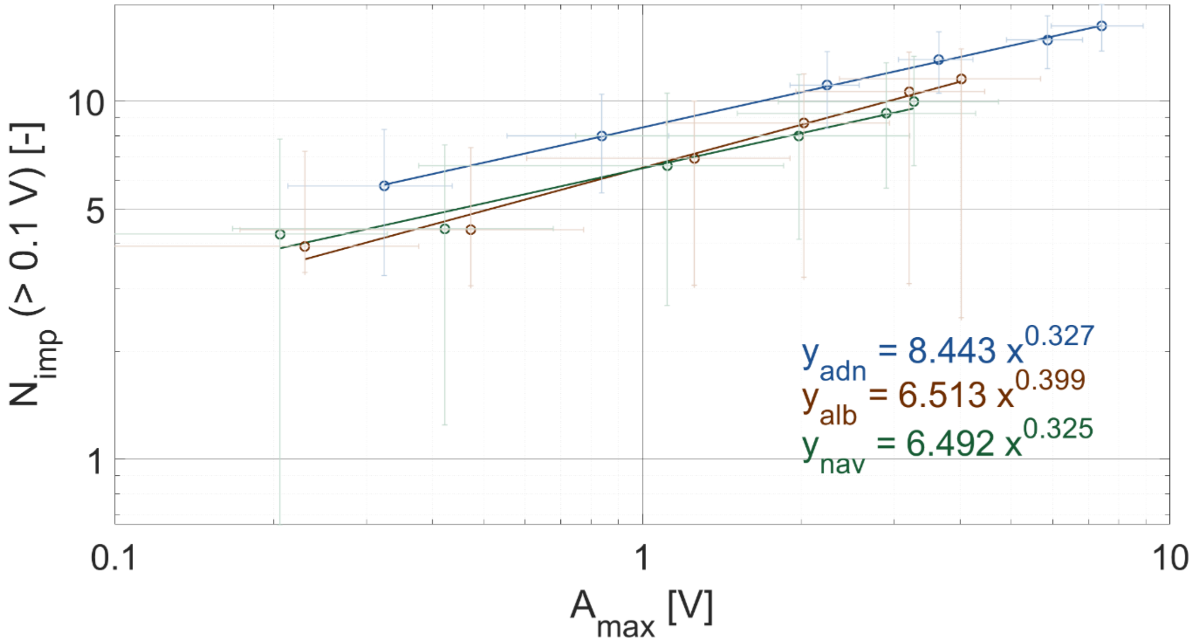

Figure S. relationship between A_{max} and N_{imp} for the experiment developed in this paper at the Avançon de Nant, Albula and Naviscence measuring stations.

Table S2. regression coefficients between the impact maximum amplitude A_{max} [V] and the associated energy propagation at one and two plates of distance recorded for each SPG at the Avançon de Nant, the Albula and the Naviscence measuring sites. The line 'All' gives the regression coefficients for all impacts on each plate taken together for a given measuring station, which are also reported in Table 7 in the main document.

	**1st plate**			**2nd plate**		
	**Intercept**	**Slope**	**R2**	**Intercept**	**Slope**	**R2**
**(a) Avançon de Nant**						
Geo1	0.175	0.950	0.980	0.075	0.905	0.985
Geo2	0.130	0.863	0.987	0.073	0.871	0.987
Geo3	0.149	0.927	0.987	0.084	0.888	0.977
Geo4	0.143	0.924	0.980	0.074	0.917	0.978
Geo5	0.192	0.939	0.986	0.082	0.876	0.987
Geo6	0.184	0.914	0.987	0.082	0.930	0.956
Geo7	0.139	0.935	0.979	0.064	0.929	0.979
Geo8	0.168	0.941	0.985	0.083	0.917	0.976
Geo9	0.143	0.923	0.976	0.089	0.859	0.989
Geo10	0.183	0.935	0.979	0.081	0.903	0.969
All	0.153	0.919	0.978	0.079	0.899	0.976
**(b) Albula**						
Geo2				0.088	0.856	0.984
Geo3				0.094	0.847	0.988
Geo4				0.088	0.795	0.978
Geo5				0.095	0.880	0.962
Geo6				0.122	0.843	0.985
Geo7				0.156	0.879	0.961
Geo8				0.131	0.871	0.926
Geo9				0.101	0.833	0.974
Geo10				0.087	0.826	0.978
Geo11				0.075	0.871	0.954
Geo12				0.087	0.874	0.982
Geo13				0.094	0.842	0.965
Geo14				0.084	0.832	0.972
Geo15				0.076	0.810	0.976
All				0.094	0.832	0.958
**(c) Naviscence**						
Geo1	0.096	0.912	0.989	0.012	0.786	0.973
Geo2	0.075	0.863	0.972	0.014	0.673	0.947
Geo3	0.082	0.870	0.975	0.013	0.742	0.948
Geo4	0.091	0.950	0.936	0.015	0.860	0.922
Geo5	0.078	0.935	0.882	0.014	0.636	0.979
Geo6	0.053	0.858	0.901	0.016	0.917	0.987
Geo7	0.088	1.009	0.942	0.020	0.792	0.968
Geo8	0.096	0.930	0.957	0.011	0.879	0.949
Geo9	0.110	1.050	0.896	0.014	0.930	0.905
Geo10	0.121	0.786	0.938	0.014	0.801	0.967
Geo11	0.092	0.940	0.967	0.014	0.661	0.961
Geo12	0.089	0.942	0.909	0.013	0.946	0.928
All	0.091	0.919	0.932	0.014	0.813	0.903

References

1. Rickenmann, D., Turowski, J.M., Fritschi, B., Wyss, C., Laronne, J., Barzilai, R., Reid, I., Kreisler, A., Aigner, J., Seitz, H. Bedload transport measurements with impact plate geophones: comparison of sensor calibration in different gravel-bed streams. *Earth Surf. Process. Landforms* **2014**, *39*, 928–942.

© 2020 by the authors. Submitted for possible open access publication under the terms and conditions of the Creative Commons Attribution (CC BY) license (http://creativecommons.org/licenses/by/4.0/).
